# The psychedelic psilocybin and light exposure have similar and synergistic effects on gene expression patterns in the visual cortex

**DOI:** 10.1186/s13041-025-01191-0

**Published:** 2025-03-18

**Authors:** Ram Harari, Dmitriy Getselter, Evan Elliott

**Affiliations:** Azrieli Faculty of Medicine, Bar Ilan University, Safed, Israel

## Abstract

Psilocybin, a psychedelic compound found in specific hallucinogenic mushrooms, is known to induce changes in visual perception and experience in humans. However, there is little knowledge of the molecular mechanisms through which psilocybin affects vision-associated regions in the brain, such as the visual cortex. The current study determined both psilocybin-induced and experience-dependent changes (exposure to light) in visual cortex gene expression in mice. Of great interest, psilocybin induced robust gene expression changes in the visual cortex that closely mirror light-induced gene expression changes, even when the mice are kept in the dark. These gene expression changes correspond to specific molecular pathways, including synaptic functioning, and represent genes expressed in specific subtypes of neurons. In addition, exposure to both psilocybin and light induced synergetic changes in genes involved in epigenetic programming. Overall, the study determined that psilocybin induces robust changes in gene expression in the visual cortex that may have functional consequences in visual perception both in the absence and in synergy with visual experience.

**Supplementary Information:**

The online version contains supplementary material available at 10.1186/s13041-025-01191-0.

## Introduction

Psychedelic compounds, including psilocybin, are known to induce changes in visual perception and experience. Changes in visual perception often include visual hallucinations or changes in the color or geometric pattern in the visual field [1–3]. Psilocybin is the main psychedelic compound in “magic mushrooms”. Psilocybin is metabolized in the liver to psilocin, which is a potent agonist of the 5-HT2A receptor [4]. Previous studies have shown that psychedelic effects are modulated by this specific receptor [5], although other effects may be modulated through separate mechanisms, such as activation of the TrkB receptor [6].

Several human studies have found that psilocybin may directly affect the function of the visual cortex. Electrophysiology studies have found that psilocybin modulates visually-evoked potentials [7]. A separate study suggested that psilocybin increases self-inhibition of visual areas in the cortex in an eyes-closed task [2]. Therefore, psilocybin may influence function of the visual cortex both during visual sensory excitation and during rest periods. Nonetheless, it is not clear what is the relationship between psychedelic-induced regulation of the visual cortex and experience-dependent activation of the visual cortex. It is not clear whether these processes are similar, opposite, or synergistic.

Previous studies in mice have characterized light-induced gene expression changes in the visual cortex of mice [8, 9]. Light-induced changes is considered a model of experience-induced gene expression in the visual cortex. In the current study, we have determined light-induced gene expression alterations as well as psilocybin-induced gene expression alterations in the visual cortex and compare between the effects of light activation and psilocybin on visual cortex gene expression. Our results demonstrate that light and psilocybin have very similar effects on gene expression in the visual cortex and have some restricted synergistic effects on genes related to epigenetic control of gene transcription.

## Results and discussion

**Fig. 1:**
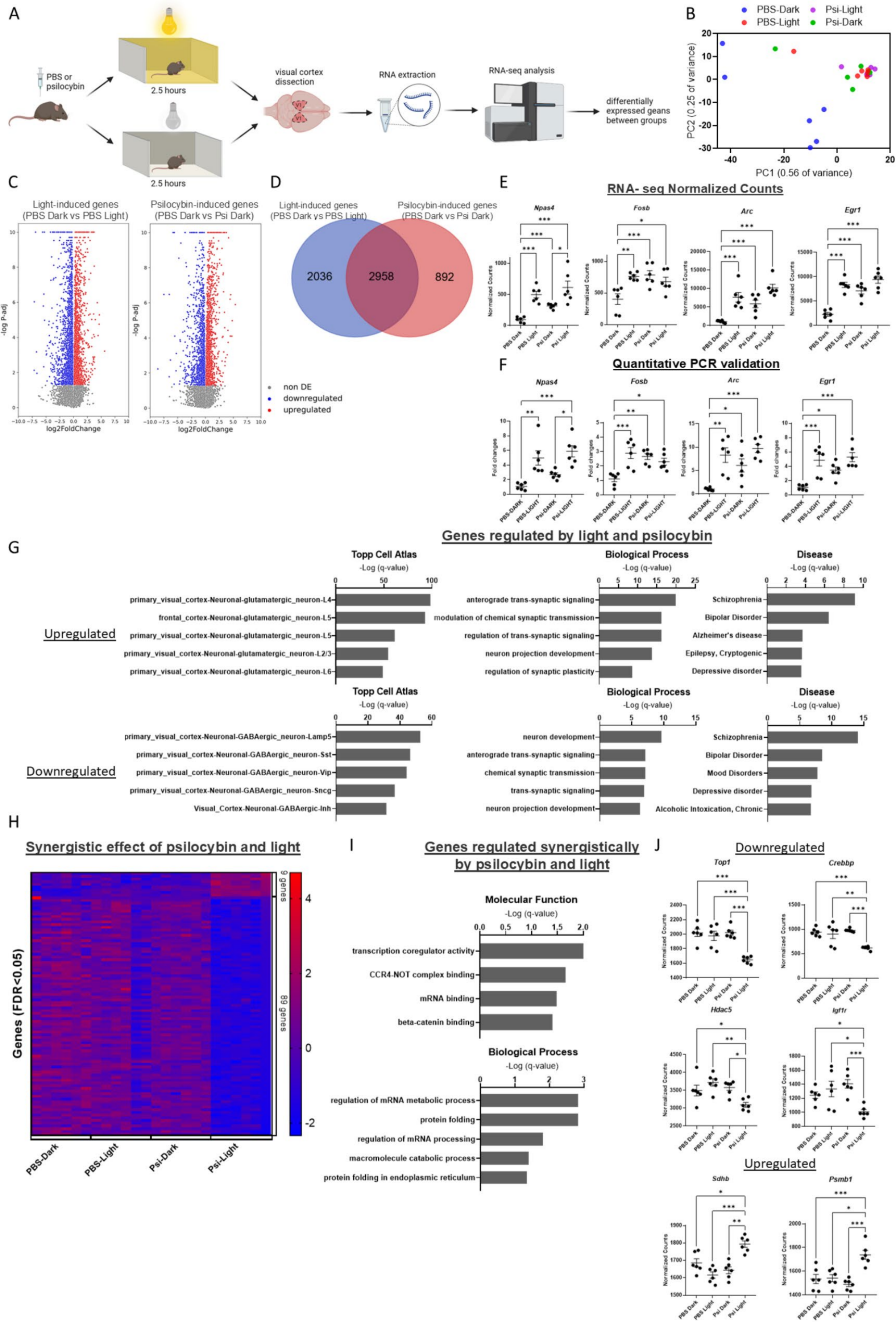
Changes in gene expression in the visual cortex under the influence of psilocybin and/or visual sensory excitation. **A**.) Schematic representation of experimental design, created with BioRender.com. **B**.) Principal Component Analysis (PCA) representing gene expression in all experimental groups (*n* = 6). **C**.) volcano plot representing gene expression changes in the light-induced genes (PBS Dark vs. PBS Light) and psilocybin-induced genes (PBS Dark vs. Psi Dark) treatment. **D**.) Venn diagram representing overlapping changes in gene expression of light-induced genes and psilocybin-induced genes (adjusted *q*-value < 0.05). **E**.) Graphs of representative genes in which gene expression significantly changed due to both psilocybin and light exposure separately. Values represent mean ± SEM, *n* = 6, ∗/∗∗/∗∗∗FDR < 0.05/0.01/0.001 (Deseq2 Wald test with correction for multiple tests). **F**.) Validation experiment of RNA-seq analysis by Quantitative PCR analysis on representative genes in the visual cortex in which gene expression significantly changed due to both psilocybin and light exposure separately. Values represent mean ± SEM, *n* = 6, ∗/∗∗/∗∗∗*p* < 0.05/0.01/0.001 by one-way ANOVA, followed by Tukey’s multiple comparisons test. **G**.) Gene Ontology (GO) enrichment analysis for genes regulated by both light and psilocybin. Gene ontology categories with FDR < 0.05 are shown. **H**.) Heatmap representing genes significantly changed in mRNA expression due to synergistic effect of psilocybin and light. **I**.) Gene Ontology (GO) enrichment analysis for genes regulated by psilocybin-light additive effect. Gene ontology categories with FDR < 0.05 are shown. **J**.) Graphs represent genes in which mRNA expression significantly changed due to the synergistic effect of psilocybin and light exposure. Values represent mean ± SEM, *n* = 6, ∗/∗∗/∗∗∗FDR < 0.05/0.01/0.001 (Deseq2 Wald test with correction for multiple tests).

C57 male mice were housed in reverse-light cycle and experimentation took place in the dark cycle. The mice were split into four experimental groups: mice placed acutely into light for 2.5 h (light group), mice kept in dark (dark group), mice treated with 5 mg/kg psilocybin, followed by placing in light (psilocybin-light group), mice treated with 5 mg/kg psilocybin and kept in the dark (psilocybin-dark group). Two and a half hours after placement of mice in the light (or maintaining in dark), mice were sacrificed, and the visual cortex was removed. RNA-seq analysis was then performed on RNA extracted from the visual cortices (Fig. 1A).

Principle Component analysis of RNA-seq data reveals that both psilocybin groups and the light groups cluster separately from the dark-no psilocybin group (control group) (Fig. 1B). Considering that the dark group is the group that maintained their normal light cycle, the light group represents light-induced changes in the visual cortex transcriptome. The fact that psilocybin-dark group clusters with the light group suggests that psilocybin induced transcriptome changes are similar to light-induced changes.

Differential expression analysis determined 4,994 genes that were differentially expressed between the dark group and the light group (light-induced genes) (Supplementary Table 1). In comparison, 3850 genes were differentially expressed between the dark group and the psilocybin-dark group (psilocybin-induced genes) (Fig. 1C). A total of 2,958 Genes overlapped between these two data sets (hypergeometric test *p* = 8.64e-1257). All of the genes that overlapped were changed in the same direction by light or psilocybin, compared to the dark group. Therefore, 76.8% of genes that were modified by psilocybin are found to also be modified by light experience, demonstrating a similar transcriptional response to both stimuli (Fig. 1D).

To gain more insight into transcriptional changes, we looked at transcriptome of specific genes that are known to be increased by light in the visual cortex. *Npas4*, *Fosb*, *Egr1 and Arc* have previously been identified in multiple studies as activated in the visual cortex upon placement of animals in light [9, 10]. In the current study, we not only find that these genes are increased by light, but we found that these genes are also increased independently by psilocybin. The combined exposure of psilocybin and light had no significant increase compared to each one alone (Fig. 1E). Furthermore, to validate our RNA-seq findings, we replicated this experiment and performed Quantitative PCR on these same genes in the visual cortex. The same general changes found in our original experiment were also found in the validation experiment (Fig. 1F). To decipher if these gene expression changes would be detected in all brain regions, we performed Quantitative PCR on thalamic samples from the same mice. The thalamus was chosen because it is the main relay of sensory information and subregions have previously been found to be responsive to light at the gene expression level [11]. There was no difference in gene expression levels among the experimental groups in the thalamus (Supplementary Fig. 1). Therefore, the effects of psilobycin and light on gene expression is at least partially specific to visual cortex.

Gene ontology analysis of genes that were upregulated by both light and psilocybin (overlapped genes) revealed that these genes are found specifically in glutaminergic neurons of all cortical layers. In comparison, genes downregulated by both light and psilocybin are found specifically in several types of GABAergic neurons. Therefore, psilocybin, and light, specifically upregulate gene expression in glutaminergic neurons and downregulate gene expression in GABAergic neurons. Both upregulated genes and downregulated genes were enriched for genes with synaptic functions and genes involved in schizophrenia and bipolar disorder (Fig. 1G).

We hypothesized that there may be some genes that are synergistically affected by light and psilocybin, and are therefore significantly downregulated or upregulated specifically in the light + psilocybin group. 98 genes found to be specifically and significantly changed in the psilocybin + light group, including 89 downregulated genes and 9 upregulated genes (Fig. 1H). Gene ontology analysis of synergistic genes showed an enrichment of genes involved in transcription coregulator activity and regulation of mRNA metabolic processes, among other categories (Fig. 1I). Examples of transcription coregulator genes that were influenced synergetically by psilocybin and light are *Top1*, *Crebbp* and *Hdac5* (Fig. 1J). TOP1 has previously been shown to be important for genome stability in neurons and can specifically regulate synapse-related genes [12]. The complex of CREB and CREBBP are well known to be crucial in regulation of synaptic plasticity [13]. HDAC5, an additional epigenetic regulator of synaptic plasticity, was previously found to regulate the expression of Arc in the visual cortex [14]. In addition, *Igf1r*, a known regulator of visual cortex function, is downregulated [8]. Upregulated genes include *Sdhb* and *Psmb1*.

Together, these findings suggest that psilocybin and light exposure have very similar effects on gene expression in the visual cortex. Light exposure is commonly used to induce neuronal activity in the visual cortex, and is considered a model of inducing experience-dependent gene expression. These findings indicate that psilocybin has a robust effect on gene expression in the visual cortex which is likely to lead to direct effects on visual experience.

It is of great interest that psilocybin had different effects on genes specifically expressed in glutaminergic neurons (upregulation of genes) compared to those expressed in GABAergic neurons (downregulation of genes). There is little previously known explanation regarding specific effects of psilocybin in specific neuronal subtypes. However, considering that glutaminergic neurons are generally excitatory, the current results suggest that psilocybin promotes excitatory activity in the visual cortex. The cell-type specific effects of psilocybin may be brain site specific, as a recent manuscript determined increases in GABAergic activity in the amygdala under the influence of psilocybin [15]. A limitation of the current work is that this is bulk RNA-seq and not single cell RNA-seq. Future single cell analysis are necessary to understand the specific genes that are effected by psilocybin in specific subtypes of neurons and glia.

Many of the genes that were influenced synergistically by light exposure under the effects of psilocybin (psilocybin-light group) include transcription regulators, such as *Top1*, *Crebbp*, and *Hdac5* (Fig. 1J). The decreased expression of these transcription regulators may have more long term effects on gene transcription and protein translation in the primary visual cortex. For example, it has previously been shown that HDAC5 binds ARC and ERG1, a neuronal activity-related genes, inhibits their expression in the visual cortex [14]. As such, decreased *Hdac5* expression may potentiate increases in activity-related genes. Therefore, future studies can decipher more long-term influences of psilocybin in the visual cortex and their functional consequences.

In the current study, *Igf1r* is specifically decreased in mice that are exposed to light under the influence of psilocybin. *Igf1* has previously been shown to be expressed specifically in VIP-expressing inhibitory neurons in the visual cortex [8]. IGF1 works cell-autonomously on the VIP-expressing neurons and promotes their inhibition leading to decreased visual acuity. Therefore, this could lead to positive effects on visual acuity. While there are few direct studies on visual acuity in humans after psilocybin exposure, studies have indicated an increase in ability to distinguish colors in color-blind individuals [16]. Overall, this study shows that psilocybin has robust effects on gene expression in the visual cortex that are similar, and sometimes synergistic, to light-induced gene expression patterns.

## Electronic supplementary material

Below is the link to the electronic supplementary material.

Supplementary Material 1

Supplementary Material 2

Supplementary Material 3

Supplementary Material 4

Supplementary Material 2

## Data Availability

RNA-seq sequencing reads supporting the conclusions of this article is included within the additional file and the raw RNA-seq dataset supporting the conclusions of this article is available in the Gene Expression Omnibus repository (GSE285605).
