# Supplementary material for: The psychedelic psilocybin and light exposure have similar and synergistic effects on gene expression patterns in the visual cortex
Source: Mol Brain. 2025 Mar 18;18:23. doi: 10.1186/s13041-025-01191-0 (PMC11921621; doi:10.1186/s13041-025-01191-0)
Supplement: Supplementary file 2 — Supplementary Material 2 [file 13041_2025_1191_MOESM2_ESM.docx]

**Methods**

**Animals**

8-10 weeks old C57BL/6J male mice (supplied by Invigo Laboratories, Rehovot, Israel) were housed according to Federation of Laboratory Animal Science Associations (FELASA) guidelines. Mice were maintained in a vivarium at 22 C with a reversed light-cycle (lights on at 19:00 hours, off at 07:00 hours). Food and water were provided ad libitum. Littermate mice were randomly selected to experimental groups. All experimentation was approved by the Institutional Animal Care and Use Committee (IACUC) under protocol number 42-06-2021.

**Pharmacological treatment of mice**

Mice were injected i.p. in a volume of 200 µl with 5 mg/kg psilocybin (4-phosphoryloxy-N,N-dimethyltryptamine) (psi-411-fb-10, Lipomed), or with vehicle of phosphate-buffered saline (PBS) (02-023-5A, Sartorius).

**Visual stimulation and brain dissection**

Experimentation took place in the dark cycle. Mice were placed in reverse light cycle room when received from Invigo Laboratories (see above, at 3 weeks of age). The experiment took place at 8 weeks of age. The reverse light cycle room has dark phase from 7 am to 7 pm. The experiment started at 12pm and ended by 2:30pm. The mice were split into four experimental groups: mice placed into light (light group), mice kept in dark (dark group), mice treated with 5mg/kg psilocybin, followed by immediate placement in light (psilocybin-light group), mice treated with 5mg/kg psilocybin and kept in the dark (psilocybin-dark group). Two and a half hours after placement of mice in the light (or maintaining in dark), mice were sacrificed by decapitation, brains were isolated, and the visual cortex (V1) was removed and stored immediately on dry ice.

**RNA isolation and library preparation**

The RNeasy Mini kit (Quigen, Ilex, Petah-Tikva, Israel, # 74004) isolation procedure was carried out according to the manufacturer’s instructions. Quality of isolated RNA was tested using the Agilent RNA Pico 6000 kit and Bioanalyzer 2100 at the Genome Technology Center of the Azrieli Faculty of Medicine, Bar-Ilan University. 350 ng of total RNA were used for mRNA enrichment using NEBNext mRNA polyA isolation module (NEB, Ornat, Rehovot, Israel, #E7490L) and libraries for Illumina sequencing were prepared using the NEBNext Ultra II RNA kit (NEB, Ornat, Rehovot, Israel, #E7770L). Quantification of the library was performed using dsDNA HS assay kit and Qubit (Molecular Probes, Life Technologies, Rhenium, Modi’in, Israel) and quantification was performed using the Agilent DNA HS kit and Bioanalyzer 2100. 10 nM of each library were pooled together and were diluted to 4 nM according to NextSeq manufacturer’s instructions. 1.1 pM was loaded into the flow cell with 1% PhiX library control.

**Bioinformatics**

Poly-A/T stretches and Illumina adapters were trimmed from the reads using cutadapt ^1^; resulting reads shorter than 30bp were discarded. Reads were mapped to the M. musculus reference genome GRCm39 using STAR ^2^, supplied with gene annotations downloaded from Ensembl (and with EndToEnd option and outFilterMismatchNoverLmax was set to 0.04). Expression levels for each gene were quantified using htseq-count ^3^, using the gtf above. Differentially expressed genes were identified using DESeq2 ^4^ with the betaPrior, cooksCutoff and independent Filtering parameters set to False. Raw P values were adjusted for multiple testing using the procedure of Benjamini and Hochberg. Pipeline was run using snakemake ^5^. Enrichment analyses for the Gene Ontology (GO) terms were performed using the online ToppGene Suite software. GO terms were considered significant when the Benjamini and Hochberg FDR adjusted p value was below 0.05. The raw data and read count data from this analysis are available at GSE285605.

**Quantitative PCR analysis**

Mice were sacrificed by decapitation, brains were removed, and amygdala and thalamus were dissected on dry ice. RNAwas synthesized using the RNeasy Mini kit (Quigen, Ilex, Petah-Tikva, Israel, # 74004) isolation procedure was carried out according to the manufacturer’s instructions. Quality of isolated RNA was tested using the Agilent RNA Pico 6000 kit and Bioanalyzer 2100 at the Genome Technology Center of the Azrieli Faculty of Medicine, Bar-Ilan University. RNA was converted to cDNA using the HighCapacity cDNA Reverse Transcription kit (Thermo Scientific, Waltham, MA, USA). Quantitative PCR for representative genes, NAPS4, FOSB, ARC and EGR1 was performed using the Fast Start Universal SYBR Green Master (Roche, Basel CH) and ViiA™7 Quantitative PCR System (Life Technologies, Carlesbad, CA, USA). The Quantitative PCR program was run for 40 cycles and included a melting temperature of 95 °C for 10 s, and an annealing temperature of 60 °C for 30 s. Relative quantification of RNA expesion was performed using the ΔΔCt method and a one-way ANOVA was performed using Graphpad prism 9.3 software. The primers used in the reactions are listed in Supplementary Table [2](https://www.nature.com/articles/s41398-023-02611-2#MOESM5).

**References**

1. Martin, M. Cutadapt removes adapter sequences from high-throughput sequencing reads. *EMBnet J* **17**, (2011).

2. Dobin, A. *et al.* STAR: Ultrafast universal RNA-seq aligner. *Bioinformatics* **29**, (2013).

3. Anders, S., Pyl, P. T. & Huber, W. HTSeq-A Python framework to work with high-throughput sequencing data. *Bioinformatics* **31**, (2015).

4. Love, M. I., Huber, W. & Anders, S. Moderated estimation of fold change and dispersion for RNA-seq data with DESeq2. *Genome Biol* **15**, (2014).

5. Köster, J. & Rahmann, S. Snakemake-a scalable bioinformatics workflow engine. *Bioinformatics* **28**, (2012).
