# Supplementary figures and images for: The psychedelic psilocybin and light exposure have similar and synergistic effects on gene expression patterns in the visual cortex
Source: Mol Brain. 2025 Mar 18;18:23. doi: 10.1186/s13041-025-01191-0 (PMC11921621; doi:10.1186/s13041-025-01191-0)

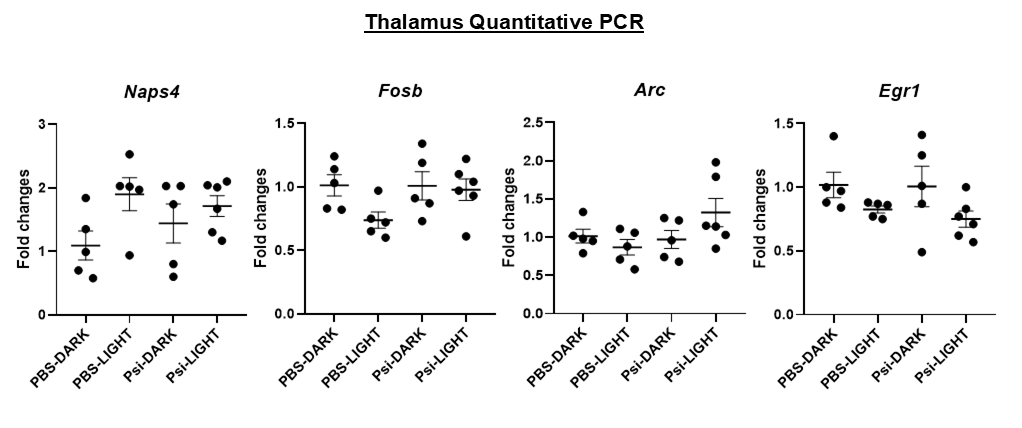

Supplement: Supplementary file 3 — Supplementary Material 3 [file 13041_2025_1191_MOESM3_ESM.png]
